# Enhanced Visible Light-Responsive Photocatalytic Properties of Ag/BiPbO_2_Cl Nanosheet Composites

**DOI:** 10.1186/s11671-018-2706-z

**Published:** 2018-09-21

**Authors:** Ai Jiao Xu, Shang Shen Feng, Shi Jie Shen, Yan Ping Liu, Wen Wu Zhong

**Affiliations:** Department of Materials, Taizhou University, Taizhou, 318000 China

**Keywords:** Photocatalysis, Co-catalyst, Hydrothermal, Bismuth-based semiconductor

## Abstract

Ag/BiPbO_2_Cl nanosheet composites were successfully prepared by hydrothermal synthesis and photo-reduction. The morphology, microstructure, and optical properties of the as-prepared Ag/BiPbO_2_Cl nanosheet composites were characterized using TEM, XRD, and UV-Vis diffuse reflection spectroscopy. The prepared Ag/BiPbO_2_Cl nanosheet composites with 0.5 wt% Ag exhibit favorable photocatalytic activity, which is 3.6 times that of BiPbO_2_Cl nanosheet. The enhanced photocatalytic properties can be attributed to the inner electromagnetic field, higher visible light response range, excellent conductivity, and lower Fermi level of Ag.

## Background

In recent years, environmental pollution has become increasingly serious. In order to solve the problem of organic pollutants, semiconductor photocatalytic materials have been widely adopted due to their unique advantages [1–4]. ZnO, TiO_2_, and other wide bandgap semiconductors are popular in photocatalytic degradation of organic pollutants [5–8]. However, wide bandgap semiconductors can only absorb ultraviolet lights, which limits the application prospects of these catalysts. Therefore, it is necessary to search for photocatalytic materials that are responsive to visible lights [9, 10].

Bismuth-based semiconductor photocatalysts possess rich structural characteristics and suitable valence band positions, which can satisfy the requirements of organic matter decomposition [11, 12]. Among them, BiPbO_2_Cl is considered to be commendable due to its narrow band gap, built-in electric field between [BiPbO_2_] and [Cl] plates, and hybrid band structure [13, 14]. Nevertheless, the fast electron-hole recombination rate limits its application in the field of photocatalysis.

It has been reported that the combination of semiconductor photocatalytic materials with noble metals or graphene can improve their photocatalytic properties [15, 16]. This is because the recombination rate of photo-generated electrons and holes decreases after compounding. Noble metals, such as Au, Ag, and Pt, have been used as electron acceptors to separate the photo-generated electron and holes [17, 18].

In this paper, the Ag/BiPbO_2_Cl composite photocatalyst was synthesized by hydrothermal method and photo-reduction for improving the photocatalytic properties of BiPbO_2_Cl nanosheets. The prepared Ag/BiPbO_2_Cl nanosheet composites with 0.5 wt% Ag exhibit favorable photocatalytic activity, which is 3.6 times that of BiPbO_2_Cl nanosheet.

## Methods

### Preparation of Ag/BiPbO_2_Cl Nanosheet Composites

The BiPbO_2_Cl nanosheets were prepared through one-step hydrothermal method as we used before [13]. The Ag/BiPbO_2_Cl composites were synthesized by photo-reduction. The obtained BiPbO_2_Cl (1 mmol) was dispersed in 20 mL deionized water with the aid of magnetic stirring, and then, an appropriate amount of AgNO_3_ was added. The suspension was then irradiated with a 500-W Xe lamp with stirring at room temperature for 3 h, with light being cut off below 420 nm using a cut-off filter. The resulting granules were washed with deionized water to remove residual organic matter and dried in air at 80 °C for 2 h. In order to study the effect of Ag content on the photocatalytic activity of BiPbO_2_Cl, the added contents of Ag were denoted as 0.25, 0.5, and 0.75 wt%.

### Photocatalytic Activities

The photocatalytic activity was characterized in a XPA series photochemical reaction instrument by a 500-W Xe lamp with a cutoff filter of 420 nm. The characterization of photocatalytic activity of samples was used by methyl orange (MO) as organic dyes. During the photocatalytic performance test, 50 mg Ag/BiPbO_2_Cl nanosheet composite powders was added into 50 mL MO aqueous solution (10 mg/L) with continuous stirring for 1 h in the dark. The absorption spectra of the solution were collected on a Shimadzu UV-2700 spectrometer.

### Sample Characterization

The powder’s X-ray diffraction (XRD) patterns were measured on a PANalytical X’Pert Pro X-ray diffractometer with Cu Kα radiation (1.54178 Å). The surface morphology was obtained on the scanning electron microscope (SEM, Hitachi S-4800). Transmission electron microscope (TEM) morphology was measured on a JEOL JEM-2011 TEM. The UV-vis diffuse reflectance spectra were measured on Shimadzu UV-2450. The X-ray photoelectron spectroscopy (XPS) was measured on a Pekin Elmer PHI-5300 XPS. The photoluminescence (PL) emission spectra were measured on a Shimadzu RF-5301 with excitation wavelength at 320 nm.

## Results and Discussion

**Fig. 1:**
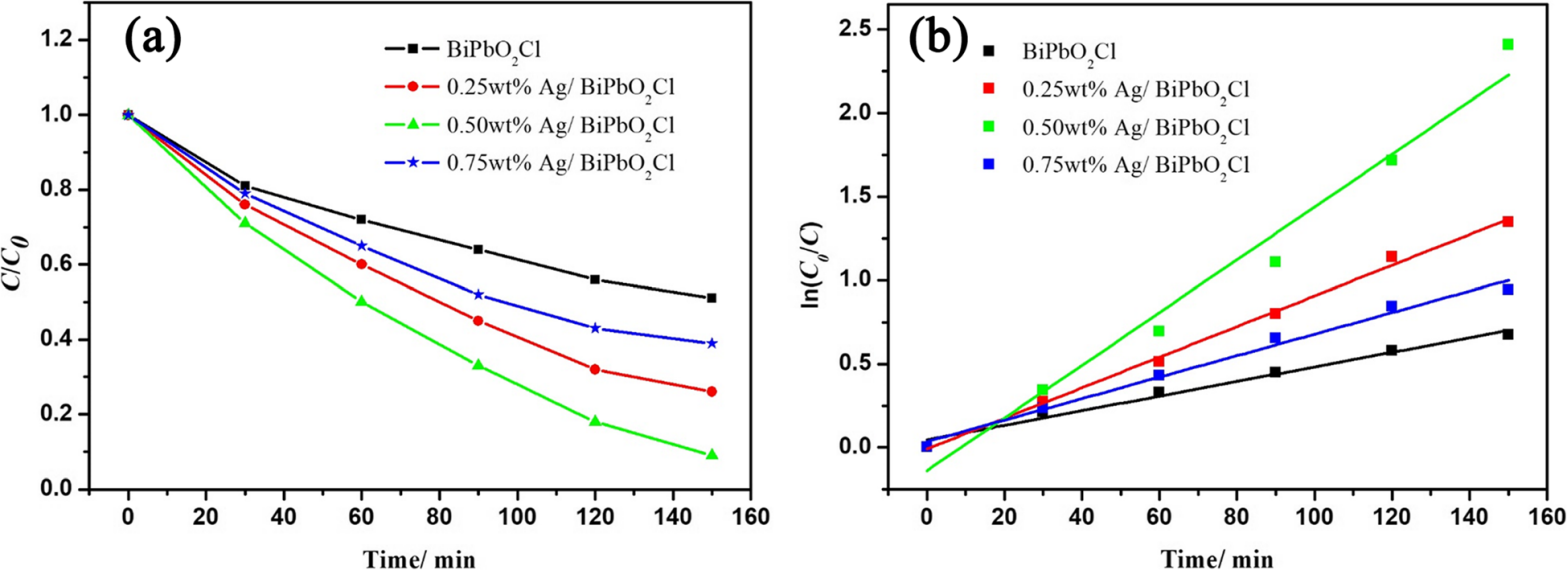
**a** Photocatalytic degradation of MO with BiPbO_2_Cl and Ag/BiPbO_2_Cl composites. **b** Kinetics of MO decolorization in solutions

Photocatalytic activity of the BiPbO_2_Cl and Ag/BiPbO_2_Cl composites has been evaluated with degradation of MO under illumination of visible light (> 420 nm). The concentration of the MO liquid is characterized by the relative absorption strength at 464 nm. Figure 1a shows the visible light photocatalytic activity of the BiPbO_2_Cl and Ag/BiPbO_2_Cl composites. Prior to degradation, the MO solution containing the photocatalyst was stirred for 1 h in the dark environment to achieve adsorption equilibrium. From Fig. 1a, it can be concluded that the photocatalytic efficiency of the BiPbO_2_Cl composites increases with the increase of Ag content, reaching a maximum when the Ag content is 0.5 wt%. This may be due to the absorption of photo-generated electrons by Ag, resulting in a decrease in the photo-generated electron-hole recombination rate, thereby increasing its photocatalytic activity. As the Ag content further increases, its photocatalytic efficiency decreases. When the content of Ag further increases, the content of BiPbO_2_Cl correspondingly decreases, resulting in a decrease in the number of photo-generated carriers and so as the photocatalytic activity. Figure 1b shows the photocatalytic reaction kinetics of the BiPbO_2_Cl and Ag/BiPbO_2_Cl composites. From Fig. 1b, we can draw that the MO degradation rate over Ag/BiPbO_2_Cl composites (0.0158 min^−1^) is about 3.6 times that of the BiPbO_2_Cl (0.0044 min^−1^).

**Fig. 2:**
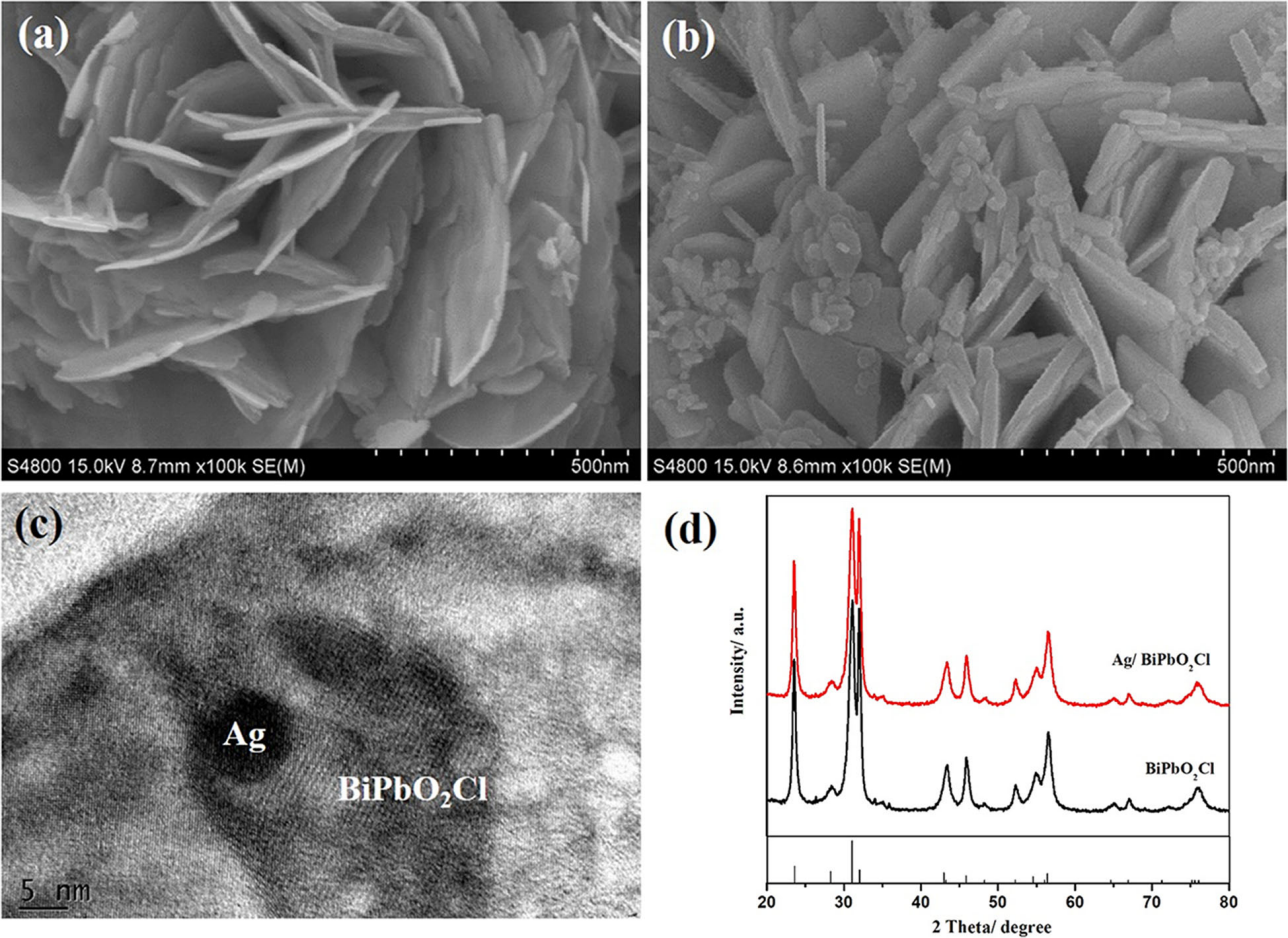
The SEM of BiPbO_2_Cl (**a**) and 0.5 wt% Ag/BiPbO_2_Cl composites (**b**). **c** High-resolution TEM image of the 0.5 wt% Ag/BiPbO_2_Cl composites. **d** XRD of samples

**Fig. 3:**
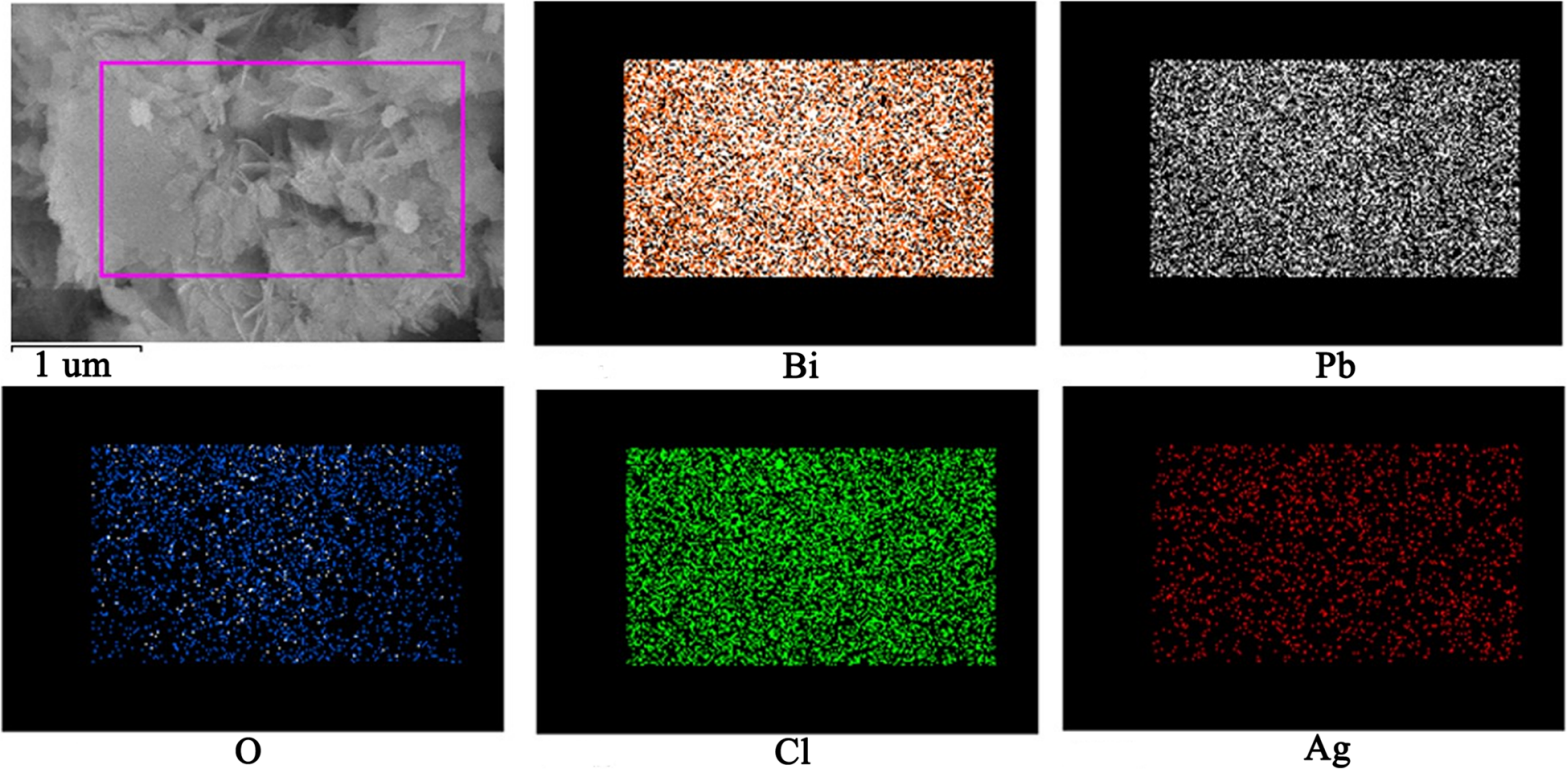
EDS mapping of element of Ag/BiPbO_2_Cl composites

In order to study the morphology and microstructure, SEM, TEM, and XRD were adopted for studying the BiPbO_2_Cl and Ag/BiPbO_2_Cl composites. From Fig. 2a, one can see that BiPbO_2_Cl is featured as nanosheets, with thickness of about 12 nm. Figure 2b shows the SEM morphology of 0.5 wt% Ag/BiPbO_2_Cl composites; the silver nanoparticles are randomly distributed on the surface of the nanosheet BiPbO_2_Cl. The diameter of Ag particles is about 10 nm. The HRTEM (Fig. 2c) images also reveal the existence of Ag. The existence of Ag is further evidenced by XPS. Figure 2d shows the XRD of BiPbO_2_Cl and 0.5 wt% Ag/BiPbO_2_Cl composites. Compared with the XRD pattern of the BiPbO_2_Cl, the pattern of Ag/BiPbO_2_Cl composites has no obvious changes, which may result from the low amount of Ag. The compositional analysis is measured by EDS (Fig. 3). Bi, Pb, O, Cl, and Ag elements are observed in the sample. Moreover, the EDS elemental mappings indicate that Ag element is evenly distributed throughout Ag/BiPbO_2_Cl composites.

**Fig. 4:**
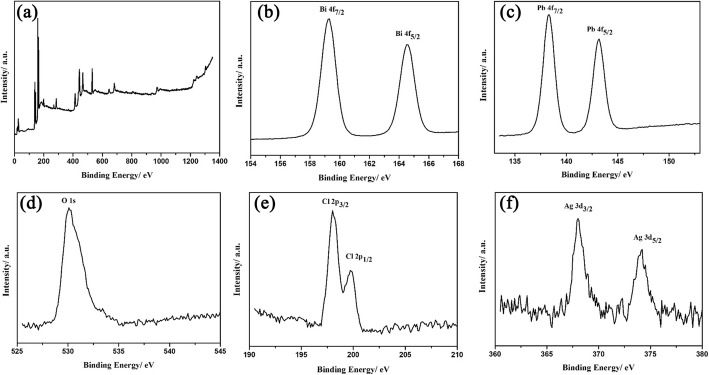
The XPS spectra of Ag/BiPbO_2_Cl composites. **a** Survey, **b** Bi 4f, **c** Pb 4f, **d** O 1s, **e** Cl 2p, and **f** Ag 3d

In order to study the surface chemical state of the sample, the XPS analysis was adopted for studying the Ag/BiPbO_2_Cl composites. As shown in Fig. 4a, the presence of Bi, Pb, O, Cl, and Ag could be observed in the XPS spectrum. As shown in Fig. 4b, the peaks of Bi 4f_7/2_ and Bi 4f_5/2_ are located at 159.1 and 164.5 eV, respectively, which are consistent with the characteristic of Bi^3+^ [19, 20]. The peaks of Pb 4f_7/2_ and Pb 4f_5/2_ are located at 137.9 and 142.8 eV (Fig. 4c), which are consistent with the characteristic of Pb^2+^ [21]. The peak of O 1s is located at 529.8 eV, which belongs to O^2−^ from the Bi–O bond (Fig. 4d). As displayed in Fig. 4e, two peaks of Cl 2p are at 197.8 and 199.4 eV, which are consistent with the characteristic of Cl^1−^ [22]. As shown in Fig. 4f, two peaks of 368.1 and 374.3 eV are observed, which correspond to Ag 3d_3/2_ and Ag 3d_5/2_, respectively. According to the results reported by Zhang et al. [23], the peaks at 368.6 and 374.6 eV can be attributed to Ag^0^.

**Fig. 5:**
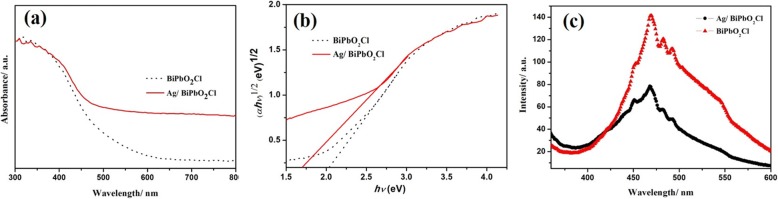
The UV-vis absorption spectra (**a**) and photoluminescence emission spectra (**b**) of BiPbO_2_Cl and 0.5 wt% Ag/BiPbO_2_Cl composites (**c**)

Compared with the yellow BiPbO_2_Cl nanosheets, the color of the Ag/BiPbO_2_Cl composites becomes darker with the increase of Ag content. The UV-vis absorption spectra of BiPbO_2_Cl and Ag/BiPbO_2_Cl composites are shown in Fig. 5a. The strong absorption below a wavelength of 600 nm is associated with the optical band gap of BiPbO_2_Cl. After loading Ag on the surface of BiPbO_2_Cl, the absorbance at the range of 450–800 nm is higher than that of pure BiPbO_2_Cl, which is due to the absorption characteristic of surface plasmon caused by the composite of Ag and BiPbO_2_Cl [24]. As a result, after the loading of Ag on the surface of BiPbO_2_Cl, the visible light response range of BiPbO_2_Cl is increased. The band gap calculated from Fig. 5a is shown in Fig. 5b. After compounding with Ag, the band gap of BiPbO_2_Cl decreases from 2.05 to 1.68 eV. In addition, the photoluminescence emission spectra of BiPbO_2_Cl and Ag/BiPbO_2_Cl composites are carried out to reflect the recombination rate of photo-generated electrons and holes. As shown in Fig. 5c, the PL intensity is decreased drastically after the loading of Ag on the surface of BiPbO_2_Cl, which is attributed to the fast transfer of photo-generated electrons from BiPbO_2_Cl to Ag, leading to reduction of recombination rate of photo-generated electrons and holes [25].

**Fig. 6:**
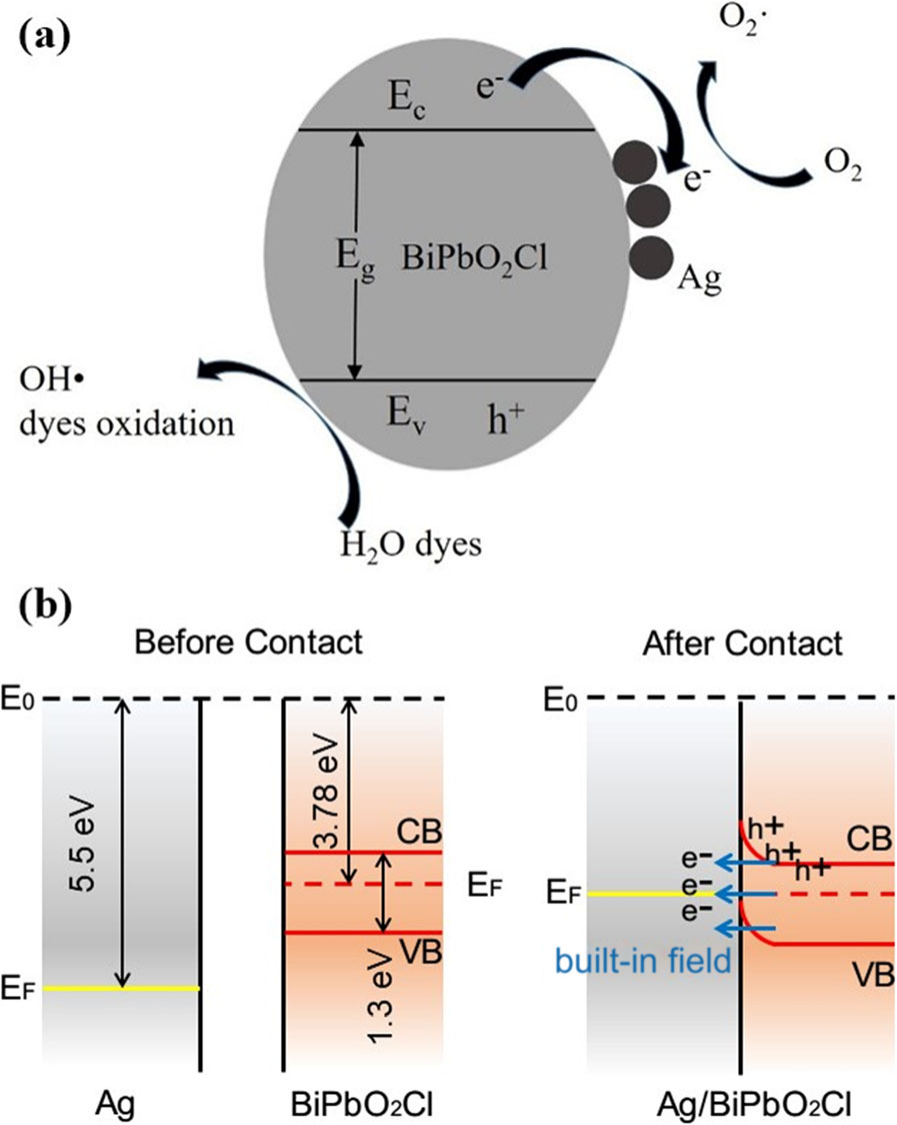
**a** Mechanistic illustration of Ag/BiPbO_2_Cl composites for photocatalytic activity. **b** Band structure at the interface of Ag and BiPbO_2_Cl. The data used for BiPbO_2_Cl are from reference [26]

The principle of high photocatalytic activity for Ag/BiPbO_2_Cl composites is as follows. First of all, the visible light response range is increased by the composition of Ag and BiPbO_2_Cl. Secondly, the loading of Ag on the surface of BiPbO_2_Cl could generate the inner electromagnetic field. When the BiPbO_2_Cl semiconductor surface is in contact with Ag, redistribution of carriers is realized. Since the Fermi level of Ag is lower than that of BiPbO_2_Cl [26], the photo-excited electrons transfer from the BiPbO_2_Cl to Ag particles until their Fermi level is the same, thus forming the built-in field, as shown in Fig. 6b. The photo-generated electron will transfer quickly from BiPbO_2_Cl to Ag with the help of the inner electromagnetic field and excellent conductivity of Ag. Thirdly, as shown in Fig. 6a, the electrons generated by BiPbO_2_Cl will reduce the molecular O_2_ to form the O_2_• active species [27]. On the other side, the photo-generated holes tend to remain on the surface of BiPbO_2_Cl. And then, these holes will transform the water molecules on the surface of BiPbO_2_Cl into OH• active species. Under the effect of these active species of O_2_• and OH•, the MO molecules are decomposed into CO_2_ and H_2_O. These results indicate that the loading of Ag on the surface of BiPbO_2_Cl could produce high visible light photocatalytic activity.

## Conclusions

In summary, highly efficient Ag/BiPbO_2_Cl composites were prepared by hydrothermal synthesis and photo-reduction. The obtained 0.5 wt% Ag/BiPbO_2_Cl nanosheet composite material has better photocatalytic activity, which is 3.6 times that of BiPbO_2_Cl nanosheets. After BiPbO_2_Cl nanosheets and Ag are composited, the visible light response range increases and the electron-hole recombination rate decreases, thus improving the visible light photocatalytic properties. The excellent photocatalytic property of Ag/BiPbO_2_Cl composites are attributed to the inner electromagnetic field, higher visible light response range, excellent conductivity, and lower Fermi level of Ag.

## Abbreviations

DRS: Diffuse reflection spectroscopy
MO: Methyl orange
TEM: Transmission electron microscope
XRD: X-ray diffraction
